# Supplementary material for: The Impact of Steatotic Liver Disease on Cytokine and Chemokine Kinetics During Sepsis
Source: Int J Mol Sci. 2025 Mar 1;26(5):2226. doi: 10.3390/ijms26052226 (PMC11900930; doi:10.3390/ijms26052226)
Supplement: Supplementary file 1 [file ijms-26-02226-s001.zip › ijms-3456849-supplementary.pdf]

Supplementary Tables and Figures

The impact of steatotic liver disease on cytokine and chemokine kinetics during sepsis

Figure S1: The kinetics of routine laboratory parameters on day 1 and 5 of hospitalization in patients with and without MASLD

Figure S2: Spearman correlation correlogram of liver steatosis scores and sepsis severity

Table S1: Analysis of serum cytokine and chemokine kinetics on day 1 and 5 of hospitalization in patients with and without MASLD

Table S2: Correlation analysis of steatosis grade (measured by CAP and FAST score) and serum concentrations of measured cytokines and chemokines

Table S3: Serum cytokine and chemokine concentrations in sepsis survivors and non-survivors with and without MASLD.

Supplementary Table S1. Analysis of serum cytokine and chemokine kinetics on day 1 and 5 of hospitalization in patients with and without MASLD. Shown are medians with IQRs. Wilcoxon rank sum test was performed to analyze the difference in time within the groups and Mann Whitney test between the groups.

	MASLD			Non-MASLD			MASLD vs non-MASLD	
**pg/mL**	**1. DAN**	**5. DAN**	p-value<br>difference<br>(95% CI)	**1. DAN**	**5. DAN**	p-value<br>difference<br>(95% CI)	**1.DAN**<br>p-value<br>difference<br>(95% CI)	**5. DAN**<br>p-value<br>difference<br>(95% CI)
IL-1β	7.8 (4.8-19)	8.5 (5.2-16)	0.6894<br>0.10<br>(-1.4 do 2.2)	7.3 (4.6-17)	7.2 (4-12)	0.1763<br>1.3<br>(-1.5 do 3.0)	0.5557<br>0.44<br>(-1.4 do 2.6)	0.1988<br>1.3<br>(-0.74 do 3.4)
IFN-γ	24 (15-57)	31 (20-54)	0.4332<br>1.5<br>(-1.5 do 5.0)	28 (16-53)	23 (14-36)	0.0254<br>3.7<br>(0 do 6)	0.9927<br>-4.1<br>(-7.3 do 7.3)	0.0302<br>7.8<br>(0.66 do 14.0)
TNF-α	27 (22-44)	28 (21-40)	0.3688<br>-0.7<br>(-1.9 do 1.6)	26 (20-37)	29 (20-40)	0.7485<br>-0.4<br>(-2.4 do 2.4)	0.2943<br>0.62<br>(-2.4 do 6.5)	0.9943<br>-1.2<br>(-5.1 do 5.4)
IL-6	214 (92-929)	104 (34-533)	0.0737<br>102<br>(29 do 206)	266 (97-1088)	87 (29-259)	0.0001<br>181<br>(80 do 522)	0.7296<br>-53<br>(-136 do 63)	0.2927<br>17<br>(-17 do 69)
IL-8	151 (83-394)	159 (74-392)	0.2162<br>38 (-17 do 79)	248 (137-516)	181 (92-343)	0.3883<br>30<br>(-71 do 226)	0.1070<br>-97<br>(-143 do 13)	0.6752<br>-22<br>(-71 do 54)
IL-10	70 (31-202)	30 (21-89)	0.0170<br>25<br>(3.1 do 87)	59 (41-120)	26 (10-83)	0.0767<br>19<br>(2.0 do 43)	0.7536<br>11<br>(-19 do 39)	0.1790<br>3.3<br>(-4.8 do 18)
IL-17A	11 (7.4-15)	8.1 (5.3-14)	0.7352<br>0.71<br>(-4.6 do 5.1)	5.4 (3.5-8.6)	4.7 (3-7.9)	0.1507<br>1,1<br>(-0.64 do 3.6)	0.0111<br>4.0<br>(0.77 do 6.1)	0.0104<br>2.5<br>(0.62 do 5.0)
IL-18	1605 (902-2572)	1695 (938-2880)	0.5659<br>51<br>(-161 do 388)	1426 (789-2456)	1202 (717-2859)	0.1125<br>219<br>(-55 do 38)	0.4669<br>179<br>(-246 do 530)	0.2527<br>493<br>(-143 do 673)

IL-23	53 (28-124)	34 (13-97)	0.0066<br>15<br>(0.23 do 47)	30 (17-55)	25 (11-42)	0.0017<br>6.8<br>(1.4 do 25)	0.0366<br>20<br>(0.72 do 41)	0.2473<br>6.7<br>(-4.3 do 23)
IL-33	312 (262-656)	383 (277-833)	0.0069<br>-108<br>(-219 do -29)	218 (138-391)	252 (150-432)	0.2270<br>-43<br>(-92 do 19)	0.0015<br>94<br>(52 do 182)	0.0027<br>131<br>(42 do 254)

	**MASLD**			**Non-MASLD**			**MASLD vs non-MASLD**	
**pg/mL**	**1. DAN**	**5. DAN**	p-value<br>difference<br>(95% CI)	**1. DAN**	**5. DAN**	p-value<br>difference<br>(95% CI)	**1.DAN**<br>p-value<br>difference<br>(95% CI)	**5. DAN**<br>p-value<br>difference<br>(95% CI)
IP-10<br>CXCL10	971 (462-3007)	553 (336-1082)	0.0012<br>233<br>(67 do 673)	461 (262-976)	438 (291-688)	0.2110<br>50<br>(-124 do 242)	0.0009<br>510<br>(144 do 812)	0.0725<br>117<br>(-11 do 254)
Eotaxin<br>CCL11	139 (84-225)	138 (100-289)	0.3053<br>-5.3<br>(-34 do 23)	120 (62-197)	159 (89-317)	0.0010<br>-41<br>(-103 do -3.4)	0.1433<br>23<br>(-8.3 do 54)	0.8631<br>-21<br>(-51 do 38)
TARC<br>CCL17	188 (114-390)	302 (181-722)	0.0053<br>-82<br>(-157 do -21)	141 (81-502)	185 (95-529)	0.6142<br>-2.1<br>(-56 do 16)	0.3110<br>22<br>(-27 do 71)	0.0304<br>117<br>(8.9 do 153)
MCP-1<br>CCL2	644 (365-1218)	674 (403-951)	0.4986<br>-19<br>(-136 do 297)	626 (354-1314)	534 (310-1092)	0.2105<br>112<br>(-70 do 189)	0.6399<br>18<br>(-153 do 227)	0.5814<br>140<br>(-117 do 196)
MIP-1α<br>CCL3	89 (39-207)	90 (31-199)	0.4270<br>-2.3<br>(-75 do 50)	54 (31-201)	68 (43-276)	0.0785<br>-13<br>(-50 do 9.0)	0.4168<br>35<br>(-18 do 55)	0.4942<br>22<br>(-77 do 32)
MIG<br>CXCL9	518 (208-1765)	538 (201-1703)	0.8579<br>35<br>(-96 do 217)	386 (118-2168)	364 (147-1607)	0.8400<br>10<br>(-135 do 197)	0.5128<br>132<br>(-123 do 243)	0.1779<br>174<br>(-46 do 283)
ENA-78<br>CXCL5	440 (187-705)	570 (281-900)	0.0194<br>-106<br>(-207 do -0.63)	339 (117-875)	415 (194-1042)	0.0295<br>-70<br>(-166 do 6.3)	0.3581<br>101<br>(-67 do 168)	0.2446<br>155<br>(-66 do 259)

MIP-3α<br>CCL20	66 (36-235)	80 (24-340)	0.9877<br>19<br>(-6.2 do 50)	98 (32-208)	37 (15-173)	0.0292<br>44<br>(21 do 104)	0.9308<br>-32<br>(-38 do 28)	0.0386<br>44<br>(1.2 do 84)
GROα<br>CXCL1	263 (192-460)	341 (213-791)	0.0098<br>-45<br>(-182 do 8.7)	237 (129-514)	264 (162-593)	0.2580<br>-28<br>(-70 do 17)	0.3183<br>26<br>(-34 do 99)	0.0430<br>79<br>(-12 do 174)
I-TAC<br>CXCL11	112 (74-246)	145 (69-308)	0.2798<br>-1.1<br>(-54 do 30)	99 (62-219)	139 (60-381)	0.1515<br>-10<br>(-69 do 9.3)	0.2543<br>12<br>(-14 do 43)	0.6777<br>6.6<br>(-32 do 49)
MIP-1β<br>CCL4	33 (15-58)	28 (13-60)	0.8720<br>7.1<br>(-9.8 do 15)	33 (14-53)	20 (9.7-44)	0.2374<br>6.9<br>(-4.7 do 18)	0.9478<br>0.84<br>(-8.5 do 9.2)	0.2369<br>8.0<br>(-2.9 do 15)
TGF-β1	198 (83-342)	122 (43-317)	0.0336<br>31<br>(5 do 112)	89 (25-245)	154 (67-286)	0.0139<br>-67<br>(-104 do -19)	0.0037<br>104<br>(24 do 124)	0.3603<br>-32<br>(-71 do 29)

Supplementary Table S2. Correlation analysis of steatosis grade (measured by CAP and FAST score) and serum concentrations of measured cytokines and chemokines.

	CAP				FAST score				BMI			
	Day 1		Day 5		Day 1		Day 5		Day 1		Day 5	
	r*	p-value	r	p-value	r	p-value	r	p-value	r	p-value	r	p-value
**IL-1β**	-0,07	0,22	0,07	0,24	0,05	0,30	-0,05	0,29	-0,06	0,23	0,05	0,27
**IFN-a**	0,02	0,42	0,02	0,44	0,15	0,06	0,14	0,07	-0,04	0,32	0,05	0,27
**IFN-γ**	-0,03	0,38	0,16	0,05	-0,01	0,46	0,00	0,49	-0,007	0,46	0,00	0,49
**TNF-α**	0,00	0,48	0,01	0,50	0,22	0,01	0,18	0,03	0,05	0,27	-0,03	0,36
**IL-6**	-0,04	0,36	0,06	0,29	0,11	0,14	0,13	0,09	-0,16	0,05	-0,04	0,31
**IL-8**	-0,12	0,11	0,02	0,43	0,11	0,15	0,11	0,15	-0,08	0,21	0,05	0,29
**IL-10**	-0,08	0,24	0,11	0,15	0,06	0,30	0,00	0,49	-0,07	0,25	0,04	0,35
**IL-12p70**	-0,39	<0,01	-0,06	0,37	-0,01	0,47	-0,12	0,26	-0,33	0,01	-0,17	0,17
**IL-17A**	0,25	0,02	0,39	<0,01	0,09	0,26	0,20	0,07	0,09	0,22	0,24	0,03
**IL-18**	-0,06	0,25	0,04	0,34	0,19	0,02	0,13	0,09	0,05	0,29	0,03	0,36
**IL-23**	0,26	0,02	0,18	0,06	-0,01	0,48	0,00	0,50	0,36	<0,01	0,31	<0,01
**IL-33**	0,30	<0,01	0,34	<0,01	-0,03	0,40	-0,11	0,17	0,3	<0,01	0,32	<0,01
**IP-10 (CXCL10)**	0,24	0,01	0,20	0,02	0,17	0,04	0,04	0,36	0,2	0,02	0,14	0,07
**Eotaxin (CCL11)**	0,09	0,18	-0,01	0,46	-0,04	0,34	-0,14	0,07	0,07	0,20	-0,01	0,44
**TARC (CCL17)**	0,02	0,44	0,23	<0,01	-0,17	0,03	-0,14	0,08	0,04	0,32	0,21	0,01
**MCP-1 (CCL2)**	-0,03	0,36	0,01	0,48	0,11	0,12	0,03	0,37	-0,12	0,09	0,014	0,44
**MIP-1α (CCL3)**	0,02	0,45	-0,16	0,13	0,18	0,08	0,03	0,40	0,06	0,31	0,00	0,49
**MIG (CXCL9)**	0,06	0,26	0,05	0,31	0,07	0,22	-0,02	0,43	0,07	0,22	0,01	0,46
**ENA-78 (CXCL5)**	0,10	0,14	0,14	0,07	-0,14	0,07	-0,09	0,18	0,04	0,32	0,14	0,07
**MIP-3α (CCL20)**	-0,04	0,34	0,15	0,06	0,14	0,08	0,13	0,10	-0,01	0,42	0,02	0,41
**GROα (CXCL1)**	0,11	0,12	0,15	0,05	-0,07	0,22	-0,01	0,45	0,07	0,22	0,06	0,27
**I-TAC (CXCL11)**	0,08	0,19	0,00	0,50	0,08	0,21	0,07	0,24	0,01	0,42	0,02	0,40
**MIP-1β (CCL4)**	0,00	0,49	0,08	0,21	-0,03	0,37	-0,06	0,26	0,03	0,35	0,09	0,17
**TGF-β1**	0,18	0,03	-0,16	0,05	-0,09	0,18	-0,19	0,03	0,04	0,32`	-0,07	0,24

Supplementary Figure S1. The kinetics of routine laboratory parameters on day 1 and 5 of hospitalization in patients with and without MASLD. Shown are medians with IQRs. Two-way RM-ANOVA was performed with multiple comparison analysis.

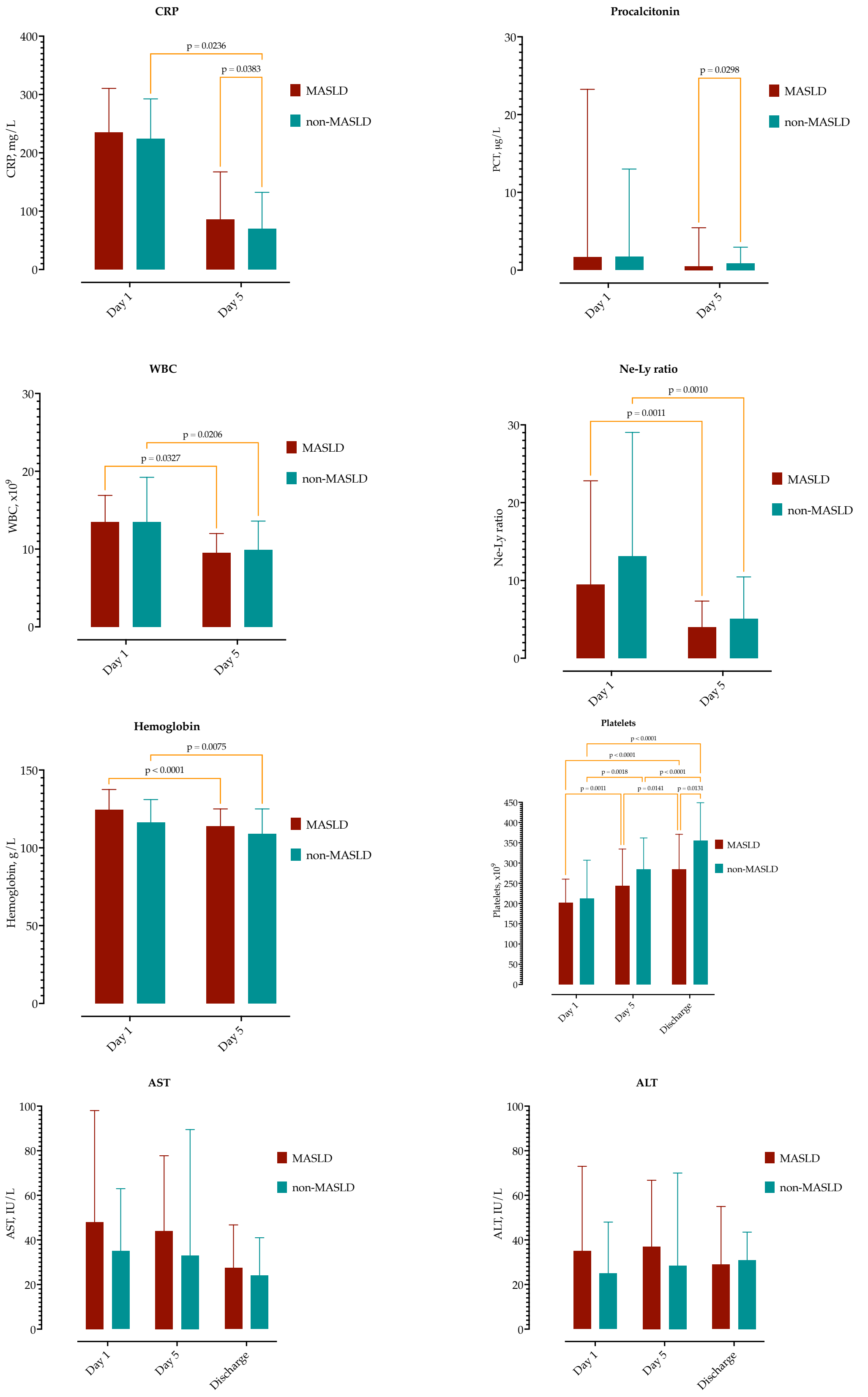

Supplementary Figure S2. Spearman correlation correlogram of liver steatosis scores and sepsis severity. The strength of the correlation between two variables is represented by the color at the intersection of those variables. Colors range from dark blue (strong negative correlation; r = -1.0) to red (strong positive correlation; r = 1.0). Shown are only significant association ($p < 0.05$).

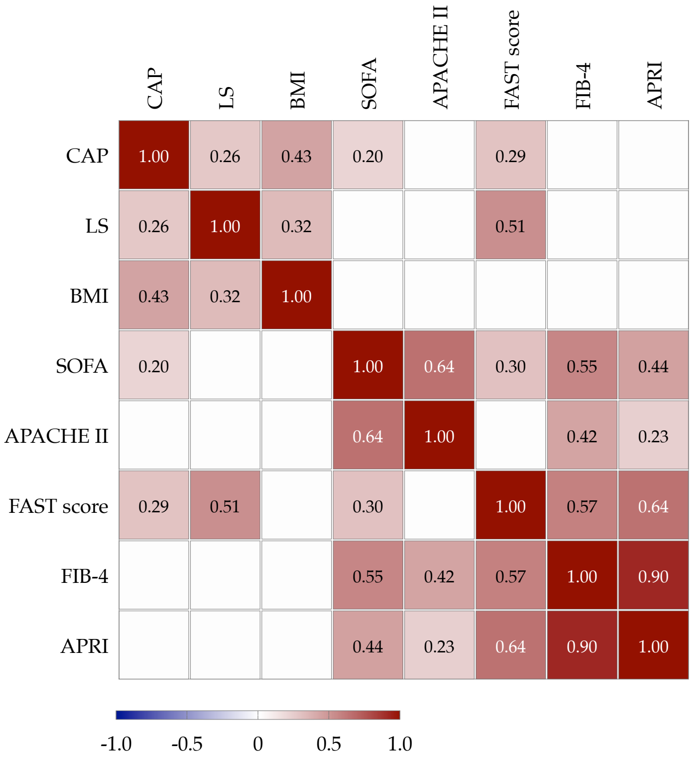

Supplementary Table S3. Serum cytokine and chemokine concentrations in sepsis survivors and non-survivors with and without MASLD.

	**MASLD died**		**MASLD survived**		**Non-MASLD died**		**Non-MASLD survived**	
	Day 1	Day 5	Day 1	Day 5	Day 1	Day 5	Day 1	Day 5
**IL-1β**	5.6 (3.5-11.5)	15.6 (6.1-44.2)	8.3 (5.1-18.5)	8.5 (4.4-16.2)	5.9 (3.1-9.5)	7.5 (4.0-20.1)	7.3 (4.7-17.7)	7.0 (3.9-12.3)
**IFN-α**	40.2 (24.5-1515.0)	34.9 (24.5-798.4)	24.5 (14.3-77.8)	24.5 (15.1-48.2)	1515.0 (18.8-15150)	26.6 (19.8-435.6)	24.5 (12.8-93.2)	24.5 (12.8-69.7)
**IFN-γ**	24.1 (15.1-38.3)	34.4 (26.1-110.4)	24.1 (16.2-73.1)	31.1(17.5-54.5)	32.1 (22.0-40.7)	24.7 (12.4-49.4)	26.2 (15.9-66.4)	22.9 (13.7-32.5)
**TNF-α**	29.9 (26.7-84.1)	25.6 (20.9-69.8)	25.2 (21.5-39.3)	29.4 (21.1-47.8)	49.0 (21.0-64.6)	36.0 (33.1-75.0)	27.0(19.7-35.9)	29.0 (19.8-41.5)
**IL-6**	153.4 (81.4-1675.2)	4602.3 (336.7-17528.5)	203.0 (87.0-535.1)	77.4 (24.1-171.7)	216.1 (45.7-763.6)	282.8 (99.2-4897.4)	301.8 (107.7-1448.4)	66.3 (20.2-171.9)
**IL-8**	147.9 (47.1-1218.0)	72.0 (19.8-1158.7)	54.9 (31.3-154.1)	54.9 (21.9-142.9)	103.1 (25.8-1082.7)	554.5 (157.1-117.1)	61.1 (53.4-184.1)	54.9 (36.8-85.6)
**IL-10**	72.9 (35.1-1279.0)	211.6 (39.2-1062.9)	50.2 (26.7-173.2)	26.0 (18.4-105.0)	62.8 (30.4-122.8)	76.8 (37.7-103.5)	56.0 (44.7-126.0)	17.8 (7.7-65.6)
**IL-12p70**	32.9 (22.1-54.6)	31.5 (14.9-47.0)	19.4 (14.0-45.6)	26.7 (12.2-56.6)	24.4 (19.4-923.8)	27.7 (23.8-31.6)	43.2 (27.0-81.3)	22.7 (12.2-28.9)
**IL-17A**	12.3 (8.2-18.8)	11.2 (8.1-15.4)	9.7 (7.1-14.1)	7.8 (5.3-13.9)	5.2 (3.0-7.6)	3.3 (3.0-8.2)	5.2 (3.6-8.4)	4.7 (3.0-7.7)
**IL-18**	2433.0 (1268.4-4981.5)	2913.8 (1349.3-5079.1)	1387.4 (886.3-2110.1)	1350.8 (921.1-2523.4)	2097.6 (523.1-2416.6)	2932.1 (724.6-4612.1)	1328.7 (804.0-2571.6)	1194.1 (693.7-2285.7)
**IL-23**	51.2 (26.3-105.7)	25.0 (14.2-42.3)	45.4 (25.7-151.5)	47.2 (13.1-111.6)	25.5 (4.0-32.8)	22.3 (9.5-51.2)	30.6 (17.4-56.8)	25.5 (12.1-38.9)
**IL-33**	275.0 (219.9-599.6)	381.0 (300.0-450.0)	329.4 (269.8-682.5)	448.6 (274.2-1012.5)	329.9 (224.4-535.4)	774.8 (142.6-878.8)	218.5 (130.8-471.3)	275.0 (151.5-432.1)
**IP-10**	1289.7 (540.4-2981.4)	632.7 (259.7-2095.9)	778.2 (409.6-3036.8)	559.7 (332.9-1332.6)	705.2 (158.8-913.2)	747.1 (403.5-1323.8)	418.5 (270.1-1072.3)	404.1 (252.0-606.5)
**Eotaxin**	95.2 (69.7-214.5)	80.4 (42.2-253.3)	139.7 (91.7-299.6)	152.3 (105.5-415.0)	143.1 (55.3-240.4)	358.0 (96.3-796.4)	111.4 (63.1-183.9)	161.2 (88.2-264.2)
**TARC**	208.9 (88.0-252.1)	199.7 (153.8-1821.0)	172.1 (125.7-488.3)	302.4 (186.1-721.5)	70.1 (58.5-90.4)	78.1 (64.6-157.9)	145.9 (114.2-494.1)	212.5 (103.4-561.2)
**MCP-1**	1276.8 (827.4-5053.2)	924.5 (556.8-8992.2)	977.4 (617.8-1748.4)	875.7 (671.0-1217.8)	661.4 (320.9-928.6)	656.3 (594.7-5348.9)	1118.5 (723.2-2276.2)	899.1 (549.1-1425.2)
**MIP-1α**	151.3 (45.3-2971.7)	96.8 (13.8-212.9)	80.8 (31.8-126.4)	94.7 (31.2-204.8)	1719.0 (148.7-3099.0)	210.0 (154.5-247.6)	49.3 (31.0-132.8)	59.7 (34.2-288.9)
**MIG**	519.8 (219.7-1487.1)	596.0(280.9-8000.0)	504.7 (182.8-2467.1)	630.6 (197.4-1703.0)	172.7 (67.2-388.8)	128.1 (88.0-155.4)	401.3 (125.3-2338.1)	390.7 (158.3-1319.0)
**ENA-78**	347.4 (209.4-646.4)	628.4 (399.8-1255.3)	548.8 (234.1-723.6)	654.7 (357.1-980.0)	107.3 (57.5-217.3)	165.7 (93.4-475.2)	361.9 (138.9-894.9)	437.1 (208.6-1090.4)
**MIP-3α**	114.3 (19.8-857.5)	187.8 (60.3-3359.0)	57.1 (36.9-207.0)	50.7 (22.2-650.6)	17.7 (9.8-33.0)	27.1 (14.8-411.9)	110.1 (37.9-243.3)	35.1 (14.5-130.4)
**GROα**	265.2 (122.9-615.8)	883.9 (251.1-5840.5)	279.0 (202.9-479.6)	389.6 (219.6-825.0)	174.8 (938-248.1)	256.4 (220.8-496.5)	245.0 (123.7-610.6)	253.5 (148.2-476.4)
**I-TAC**	102.6 (66.1-186.4)	149.1 (37.4-905.2)	117.6 (83.9-270.1)	151.6 (77.0-326.1)	80.2 (62.6-233.9)	143.2 (32.9-169.1)	99.6 (62.5-198.2)	131.7 (60.7-396.2)
**MIP-1β**	15.8 (11.2-91.1)	39.9 (31.0-147.6)	33,8 (15,9-53,9	25,0 (13,0-60,3)	5,7 (3,1-34,4)	53,5 (9,1-97,9)	25.7 (14.,5-65.8)	18,1 (9.2-42.5)
**TGF-β1**	280.3 (163.8-472.8)	125.2 (96-340.6)	156 (80-357.2)	126.2 (44-325.8)	43.6 (9.2-59.1)	111 (47.1-214.1)	108.4 (30-248.8)	153.7 (78.5-364.4)
